# Surgeon effects on cataract refractive outcomes are minimal compared with patient comorbidity and gender: an analysis of 490 987 cases

**DOI:** 10.1136/bjophthalmol-2021-320231

**Published:** 2021-11-11

**Authors:** Rachael Hughes, Petros Aristodemou, John M Sparrow, Stephen Kaye

**Affiliations:** 1 MRC Integrative Epidemiology Unit, University of Bristol, Bristol, UK; 2 School of Epidemiology and Public Health, University of Bristol, Bristol, UK; 3 Eye and Vision Science, University of Liverpool, Liverpool, UK

**Keywords:** optics and refraction, lens and zonules, treatment surgery

## Abstract

**Aim:**

To investigate effect of patient age, gender, comorbidities and surgeon on refractive outcomes following cataract surgery.

**Methods:**

Study population: patients on UK national ophthalmic cataract database on cataract operations undertaken between 1 April 2010 and 31 August 2018. Variables examined included gender, age, diabetic retinopathy, glaucoma, high myopia, inherited retinal disease, optic nerve disease, uveitis, pseudoexfoliation, vitreous opacities, retinal pathology, cataract type, previous surgery and posterior capsular rupture. A multivariate normal cross-classified model was fitted to the refractive outcome using Markov Chain Monte Carlo (MCMC) methods with diffuse priors to approximate maximum likelihood estimation. A MCMC chain was generated with a burn-in of 5000 iterations and a monitoring chain of 50 000 iterations.

**Results:**

490 987 cataract operations were performed on 351 864 patients by 2567 surgeons. Myopic and astigmatic errors were associated with posterior capsule rupture (−0.38/+0.04×72), glaucoma (−0.10/+0.05×95), previous vitrectomy (−0.049/+0.03×66) and high myopia (−0.07/+0.03×57). Hyperopic and astigmatic errors were associated with diabetic retinopathy (+0.08/+0.03×104), pseudoexfoliation (+0.07/+0.01×158), male gender (+0.12/+0.05×91) and age (−0.01/+0.06×97 per increasing decade). Inherited retinal disease, optic nerve disease, previous trabeculectomy, uveitis, brunescent/white cataract had no significant impact on the error of the refractive outcome. The effect of patient gender and comorbidity was additive. Surgeons only accounted for 4% of the unexplained variance in refractive outcome.

**Conclusion:**

Patient comorbidities and gender account for small but statistically significant differences in refractive outcome, which are additive. Surgeon effects are very small.

## Introduction

Progress has been made in reducing postoperative refractive prediction errors and in achieving spectacle independence through improvements in phacoemulsification cataract surgery and refinements in the acquisition of biometric data and intraocular lens (IOL) power formulae.^1–4^ Unintended and uncorrected postoperative spherocylindrical refractive errors, however, are not uncommon. Residual astigmatic errors, in particular, have a far greater adverse effect on unaided visual acuity (VA) or central blur than may be evident using a spherical equivalent^5–8^ and uncompensated refractive errors, particularly those containing oblique cylinder axes are especially destructive on stereopsis and vision.^9 10^ Most studies on cataract refractive outcomes use spherical equivalent as an outcome, as it is a scalar variable amenable to standard statistical analysis. Spherical equivalent is, however, an insensitive measure with potential systemic bias.^11^ Cylindrical errors are conventionally analysed separately due to their vectorial nature, but this leads to significant errors when analysed separately from sphere.^12 13^ Treating the refractive outcome as a spherocylinder^14 15^ has been shown to be an appropriate and more sensitive and specific approach for identifying refractive outliers than using the spherical equivalent or the mean absolute error of nearest equivalent sphere^16^ and/or cylinder.^17^

There are many well-recognised variables which may lead to an unintended refractive outcome following cataract surgery, such as previous refractive surgery, measurement errors in biometry and extremities of the axial length. What is not clear, however, is whether other factors such as coexistent pathology such as diabetic retinopathy and glaucoma are associated with less predictable and poorer refractive outcomes. While risk factors related to specific copathology, such as pseudoexfoliation and white cataract, have been identified as risk factors predisposing to surgical complications and poorer visual outcomes,^18 19^ this information is not available in terms of refractive outcome. Identifying and quantifying risk factors which are associated with unexpected refractive outcomes would provide surgeons with information to better inform the patient and to enable refinement of future IOL power formulae to improve refractive outcomes.

A key feature of refractive data is its multilevel structure, with each surgeon operating on many patients, and many patients receiving an operation on each eye. Dependencies between observations (due to the multilevel structure) must be appropriately modelled in order to obtain correct SEs.^20^ Ignoring such multilevel data structures can lead to incorrect inference.^20^ Multilevel modelling is a flexible approach that can model a wide range of multilevel structures and can be extended to jointly model multivariate outcomes such as the spherocylindrical refractive outcome (ie, simultaneously model its three components). This paper illustrates the application of a multilevel multivariate model to refractive data, estimated using Markov Chain Monte Carlo (MCMC) methods to identify ocular factors and comorbidities that may have significant associations with refractive outcomes following cataract surgery.

## Methods

### Study sample

Cataract surgery data were obtained through a data sharing agreement with the Health Quality Improvement Partnership who acted as the data controller for the National Ophthalmology Database cataract audit. Deidentified data were derived from 104 UK centres undertaking National Health Service (NHS) funded cataract surgery. Database analyses of this type which use deidentified data do not require ethical permission and are viewed as audit or service evaluation (see http://www.hra.nhs.uk/research-community/beforeyou-apply/determine-whether-your-study-is-research/). This study was conducted in accordance with the Declaration of Helsinki and the UK’s Data Protection Act. Analyses were based on data on cataract operations undertaken between 1 April 2010 and 31 August 2018. Patients were eligible for analysis if they were aged 18 years or older and had a cataract operation using phacoemulsification (where the primary reason for the eye operation was cataract surgery for visual improvement), preoperative keratometry measurements, an intended refractive outcome and a postoperative refraction measurement. The presence of any comorbidity was noted at surgery by ticking the relevant boxes indicating the presence of a number of concurrent diagnoses where applicable. This was an essential item of the surgery proforma and the surgical record cannot be saved unless this part was completed.

### Multilevel structure

The data had a two-way cross-classified multilevel structure.^20^ A surgeon operated on several patients and a patient either received a single cataract operation (to the left or right eye) or two cataract operations (one on each eye) predominantly on separate occasions. Among patients who received two operations, these were often conducted by different surgeons. Therefore, cataract operations for individual eyes were nested within cells of a two-way cross-classification of surgeons by patients.

### Analysis outcome

The outcome of interest was the difference between the postoperative and the expected refractive outcome, defined as the error of expected refractive outcome (EERO). This term was used rather than terms such as surgically induced refractive error or surgically induced refractive change as it is not always possible to assign the change to the surgery itself as there may be patient factors, and instrument or measurement errors. In addition, these terms have been inconsistently used in relation to one or more of the individual components of the refractive error, for example, spherical equivalent or cylinder rather than the spherocylinder as a compound number.

The data provided included the refractive target using a third generation formula selected by the surgeon. The electronic medical record highlights the most appropriate IOL power formula out of Hoffer Q, Holladay 1 or SRK/T, with respect to the patient’s axial length.^4^ The intended or refractive target was calculated as a spherocylinder^14 15^ using preoperative keratometry measurements observed closest to the date of operation and the surgeon selected intended sphere measurement as previously described.^16^ The difference between the preoperative steep (K2) and flat (K1) meridians was added to the intended spherical refractive outcome selected by the surgeon to give the intended refractive outcome as a spherocylinder.^17^ For those operations with multiple postoperative refraction measurements, a single postoperative measurement was selected based on when it was observed and the type of refraction measurement. Online supplemental appendix table 1 shows the order of preference for selection of the single postoperative measurement. For each operation, the observed measurement that satisfied the highest criterion category was selected. The data were transformed from the sphere/cylinder ×axis scale onto the three components of Long’s dioptric power matrix for a thin lens ( $f_{11},f_{12},f_{22}$ )^21^ before the difference between the intended and postoperative refraction was calculated. All cylinder powers are in positive cylinder format.

10.1136/bjophthalmol-2021-320231.supp1 Supplementary data

### Statistical analyses

A multivariate normal cross-classified model was fitted to the EERO on the dioptric power matrix scale ( $f_{11},f_{12},f_{22}$ ). At each level, the variances and covariances of the random effects could be distinct. The model was fitted using MCMC methods using diffuse priors to approximate maximum likelihood estimation.^22^ A MCMC chain was generated with a burn-in of 5000 iterations and a monitoring chain of 50 000 iterations. MCMC chains generated using different starting values were examined using MCMC diagnostic tools. Similarly, to estimate the mean and spread of the preoperative keratometry and postoperative refraction measurements, separate multivariate normal cross-classified models were fitted to the measurements on the dioptric power matrix scale. Analyses were conducted using Stata/MP^23–25^ (V.15.0; Stata, College Station, Texas, USA), command runmlwin^26^ and MLwiN software.^27^

Potential variables which might affect the EERO were classified into surgeon-level, between patient level (time-independent) and within patient-level (time-dependent patient characteristics) or eye-level variables. Of these, patient’s gender, age at the time of the operation, time of the refraction since the operation (within 3 months, between 3 and 6 months, between 6 and 12 months and more than 12 months) and the refraction type (subjective, autorefraction, focimetry, focimetry with second pair of glasses and other) were included as covariates. We also considered the following as covariates (at time of surgery): diabetic retinopathy, glaucoma, high myopia (defined as greater than −8.00D determined by the surgeon), inherited eye disease, optic nerve pathology, uveitis or synaechiae, pseudoexfoliation or phacodonesis, no fundal view or vitreous opacities, other macular pathology, other retinal pathology, other ocular copathology, brunescent or white mature cataract, previous vitrectomy, previous trabeculectomy and the occurrence of posterior capsular rupture during surgery. Covariates were selected for inclusion using backward selection with the likelihood ratio test. As the cross-classified model could not be estimated using maximum likelihood methods, we instead conducted this procedure using a nested 3-level multilevel model. Note that since the results for the fixed effects, that is, covariates’ results, were virtually identical between the cross-classified and nested three-level model, we would expect covariate selection to be the same for both types of models. A covariate was eliminated from the model if the p value from the likelihood ratio test was ≥0.01.

Patient’s gender was the only patient-level covariate (ie, a patient who received two eye operations would have the same value for gender on both occasions) that was included. All remaining covariates were eye-level covariates (eg, a patient’s age at time of surgery would differ between the two eye operations conducted on separate occasions).

The residuals of the model were examined using diagnostic plots. To improve satisfaction of the normality assumption, we excluded postoperative refraction measures where the absolute value of the sphere or cylinder was more than 10D. The final model was used to predict values of the EERO for future operations according to the comorbidities included in the final model (eg, whether the eye had an existing copathology such as glaucoma or had experienced an intraoperative complication such as posterior capsular rupture during surgery). These predicted values were generated for a single comorbidity and for relevant combinations. The monitoring chain of 50 000 parameter estimates (of the model) was used to derive a distribution of 50 000 predictions, from which prediction intervals were derived. All analyses were conducted on the dioptric power matrix scale ( $f_{11},f_{12},f_{22}$ ) and the results back transformed to the original scale (sphere, cylinder ×axis).

## Results

Of the 1 070 601 cataract operations undertaken between 1 April 2010 and 31 August 2018 in the National Ophthalmology Database sample, 466 711 did not have a returned recorded postoperative refraction measurement. Of the remaining cataract operations, 112 174 did not have preoperative keratometry measurements, 302 did not have an intended refractive outcome recorded in the database and 427 had a postoperative sphere or cylinder measurement with an absolute value greater than 10D, leaving 490 987 operations eligible for analysis.

These operations were performed on 351 864 patients by 2567 surgeons. For 71.7% of the operations (n=352 041), this was the patient’s first cataract operation. Median patient age at first eye cataract surgery was 76.6 years (IQR: 69.6–82.3 years) and 41.7% (n=146 874) of patients were male. Among the 139 123 patients who had surgery on both eyes, 177 patients had immediate sequential bilateral surgery and for the remaining 138 946 patients the median time between the operations was 3.9 months (IQR: 2.3–6.1 months). The same surgeon performed both operations for 31.3% (n=43 487) of these patients.

Among all 490 897 cataract operations, the mean postoperative refraction outcome was −0.50/0.49×5 (95% CI −0.51/0.50×5 to –0.49/0.49×4), with the spread of the observations indicated by 95% prediction interval −3.00/1.54×38 to 0.99/1.45×147. The majority of the postoperative refraction outcomes were recorded within 3 months of the operation (84.8%; n=416 429), with 7.5% (n=37 015), 4.1% (n=20 234) and 3.6% (n=17 304) recorded between 3 and 6 months, 6 and 12 months and more than 12 months after the operation respectively. For the type of refraction outcome, 51.3% (n=251 936) were subjective, 39.2% (n=192 245) were autorefraction, 7.8% (n=38 382) were focimetry, 0.3% (n=1601) were focimetry with second pair of glasses and the remaining 1.4% classified as other (n=350 cycloplegic, n=14 retinoscopy and n=6459 of unknown type).

Table 1 contains the reported copathologies and table 2 are the population-average effects of an ocular copathology, feature or intraoperative complication on the EERO (ie, the fixed effects of the cross-classified multilevel model). Being male was associated with a hypermetropic and astigmatic shift, while being 10 years older (at the time of the operation) was associated with a small hypermetropic and astigmatic shift. Having diabetic retinopathy was also associated with a hypermetropic and astigmatic shift and similarly for pseudoexfoliation or phacodonesis. Conversely, previous vitrectomy, high myopia, glaucoma or posterior capsular rupture were associated with a myopic and astigmatic shift. A history of uveitis, presence of synaechia, previous trabeculectomy other retinal pathology or the presence of a brunescent or white mature cataract had no effect on EERO.

**Table 1:** Ocular copathology, features or reported intraoperative complications in the operated eye

Out of 490 989 operations	N (%)
Glaucoma	42 983 (8.75%)
Diabetic retinopathy	27 073 (5.51%)
Other ocular copathology	22 383 (4.56%)
High myopia	21 155 (4.31%)
Brunescent/white mature cataract	19 123 (3.89%)
Other macular pathology	11 349 (2.31%)
Previous vitrectomy surgery	7553 (1.54%)
No fundal view/vitreous opacities	5839 (1.19%)
Pseudoexfoliation/phacodonesis	4638 (0.94%)
Posterior capsular rupture	4523 (0.92%)
Other retinal pathology	4340 (0.88%)
Uveitis/synaechiae	3399 (0.69%)
Previous trabeculectomy surgery	1952 (0.40%)
Diseased optic nerve	1689 (0.34%)
Inherited eye disease	530 (0.11%)

**Table 2:** Population-average effect of ocular copathology, feature or intraoperative complication on the error of expected refractive outcome, reported as compound numbers: sphere cylinder axis

	Posterior mean	95% credible interval
Women aged 77 years with no copathology nor complications*	−0.30/0.45×4	−0.32/0.46×5 to –0.29/0.45×4
Men	+0.12/0.051×91	+0.11/0.05×88 to +0.13/0.051×93
Age (per 10 years)	−0.0094/0.06×97	−0.012/0.06×96 to −0.0065/0.06×98
Diabetic retinopathy	+0.082/0.03×104	+0.070/0.03×96 to +0.093/0.04×110
Pseudoexfoliation/phacodonesis	+0.065/0.01×158	+0.036/0.01×23 to +0.086/0.03×145
Previous vitrectomy surgery	−0.039/0.03×66	−0.069/0.04×59 to −0.011/0.02×82
High myopia	−0.066/0.03×57	−0.085/0.04×55 to −0.047/0.02×62
Glaucoma	−0.099/0.05×95	−0.11/0.05×91 to −0.089/0.05×99
Posterior capsular rupture	−0.38/0.04×72	−0.42/0.05×64 to −0.35/0.03×88
Uveitis/synaechiae	−0.029/0.03×16	−0.071/0.05×28 to +0.0075/0.03×175
Previous trabeculectomy surgery	−0.076/0.07×113	−0.11/0.06×100 to −0.041/0.09×120
Other retinal pathology	−0.00074/0.03×77	−0.037/0.04×64 to +0.030/0.02×101
Brunescent/white mature cataract	+0.0026/0.01×69	−0.016/0.02×59 to +0.020/0.01×90
Time period		
Within 3 months†	–	–
Between 3 and 6 months	+0.062/0.03×160	+0.053/0.03×166 to +0.070/0.04×156
Between 6 and 12 months	+0.083/0.03×163	+0.071/0.03×171 to +0.094/0.04×158
More than 12 months	+0.12/0.05×172	+0.11/0.05×178 to +0.14/0.06×167
Refraction type		
Subjective†	–	–
Autorefractive	−0.062/0.05×19	−0.072/0.06×21 to −0.052/0.05×16
Focimetry	+0.019/0.00×180	+0.002/0.01×40 to +0.028/0.01×142
Focimetry (second pair glasses)	+0.00024/0.04×26	−0.064/0.07×35 to +0.057/0.03×180
Other	−0.15/0.04×161	−0.19/0.03×5 to −0.12/0.06×150

*Reference patient (woman aged 77 years with a subjective refraction observed within 3 months of operation).

†Reference category of that variable.

Reported in table 3 are the population-average differences in EERO between patients with two specified ocular comorbidities compared with patients without either ocular comorbidities (all else being equal). For example, the population-average difference in EERO of 77-year-old women with glaucoma was a myopic and astigmatic shift of −0.35/0.41×4 (95% CI −0.37/0.41×5 to −0.34/0.40×3). In contrast, the population-average difference in EERO of 77-year-old men with diabetic retinopathy was predominantly an astigmatic shift of −0.019/0.37×4 (95% CI −0.036/0.38×5 to −0.0011/0.37×3). The mean population difference between a man with diabetic retinopathy and a woman with glaucoma (for all ages) would be +0.30/0.031×96 (95% CI +0.29/0.031×86 to +0.32/0.035×106).

**Table 3:** Population-average of the EERO among women and men aged 77 years with no ocular comorbidities and population-average difference in EERO between patients with the specified two ocular comorbidities compared with patients without the specified ocular comorbidities, all else being equal

	Posterior mean/mean difference	95% credible interval
Population-average of EERO in reference patients		
Women aged 77 years with no ocular comorbidities*	−0.30/0.45×4	−0.32/0.46×5 to –0.29/0.45×4
Men aged 77 years with no ocular comorbidities*	−0.13/0.40×5	−0.14/0.40×5 to −0.12/0.40×4
Population-average difference in EERO		
Previous vitrectomy surgery and high myopia	−0.10/0.06×62	−0.14/0.07×58 to −0.070/0.04×68
Previous trabeculectomy surgery and glaucoma	−0.17/0.11×105	−0.21/0.10×98 to −0.14/0.13×111
Previous vitrectomy surgery and pseudoexfoliation/phacodonesis	+0.041/0.02×65	−0.0087/0.04×53 to +0.078/0.01×120
Pseudoexfoliation/phacodonesis and glaucoma	−0.024/0.04×103	−0.053/0.03×89 to +0.0017/0.05×113

Results reported as compound numbers: sphere/cylinder×axis.

*With a subjective refraction observed within 3 months of operation.

EERO, error of expected refractive outcome.

Table 4 contains the population-average difference in EERO between patients with posterior capsular rupture plus another specified ocular comorbidity compared with patients without either ocular comorbidities (all else being equal). The population-average difference in EERO between patients with posterior capsular rupture and high myopia compared with similarly aged female or male patients without either of these ocular comorbidities was a myopic shift of −0.45/0.064×66 again with a narrow 95% CI of −0.49/0.082×61 to −0.41/0.049×74.

**Table 4:** Population-average of the EERO among women and men aged 77 years with no ocular comorbidities and population-average difference in EERO between patients with posterior capsular rupture plus another specified ocular comorbidity compared with patients without posterior capsular rupture nor the other specified ocular comorbidity, all else being equal

	Posterior mean/mean difference	95% credible interval
Population-average of EERO in reference patients		
Women aged 77 years with no ocular comorbidities*	−0.30/0.45×4	−0.32/0.46×5 to –0.29/0.45×4
Men aged 77 years with no ocular comorbidities*	−0.13/0.40×5	−0.14/0.40×5 to −0.12/0.40×4
Population-average difference in EERO		
Posterior capsular rupture and brunescent/white mature cataract	−0.38/0.05×71	−0.42/0.07×64 to −0.34/0.04×83
Posterior capsular rupture and pseudoexfoliation/phacodonesis	−0.30/0.02×75	−0.36/0.05×59 to −0.26/0.02×110
Posterior capsular rupture and high myopia	−0.45/0.06×66	−0.49/0.08×61 to −0.41/0.05×74
Posterior capsular rupture and uveitis/synaechiae	−0.40/0.04×46	−0.46/0.07×46 to −0.34/0.01×46

Results reported as compound numbers: sphere/cylinder×axis.

*With a subjective refraction observed within 3 months of operation.

EERO, error of expected refractive outcome.

### Predicting the difference between the intended/expected and postoperative refractive error

To gain an understanding of the spread of the EERO among patients, the cross-classified multilevel model was used to predict an EERO for a new operation according to the presence of an ocular copathology or an intraoperative complication or the absence of any of these. Table 5 contains the corresponding 95% prediction intervals, that is, the range of likely values of an EERO for a future operation. For example, with 95% certainty, a man aged 77 years with an eye with pre-existing diabetic retinopathy will have an EERO of between −2.65/1.44×38 and +1.41/1.36×145. The prediction intervals were similar for all ocular copathologies, except for posterior capsular rupture which had a slightly wider prediction interval, that is, −3.18/1.47×38 and +0.90/1.34×146 for a woman and −3.02/1.45×39 and +1.04/1.33×145 for a man both aged 77 years. Note the myopic shift of the prediction interval for women compared with a men. Prediction intervals were similarly generated for the presence of more than one ocular pathology when these were likely to coexist, for example, high myopia and previous vitrectomy or glaucoma and pseudoexfoliation (table 5) and following posterior capsule rupture in the presence of other ocular pathology, for example, posterior capsule rupture and pseudoexfoliation (table 5). This gave a slightly wider prediction interval than the presence of a single copathology.

**Table 5:** 95% prediction intervals in which a future observation of the error of expected refractive outcome will fall (with 95% certainty) in men and women aged 77 years

	Men aged 77 years* 95% prediction interval	Women aged 77 years* 95% prediction interval
None	−2.65/1.45×38 to +1.41/1.35×145	−2.82/1.46×37 to +1.23/1.38×146
Diabetic retinopathy	−2.55/1.42×39 to +1.49/1.35×144	−2.70/1.44×38 to +1.34/1.38×145
Glaucoma	−2.74/1.42×39 to +1.33/1.36×144	−2.89/1.43×38 to +1.17/1.36×145
High myopia	−2.71/1.46×39 to +1.36/1.33×145	−2.87/1.49×38 to +1.19/1.35×146
Uveitis/synaechiae	−2.68/1.46×38 to +1.39/1.35×146	−2.84/1.49×37 to +1.23/1.36×147
Pseudoexfoliation/phacodonesis	−2.57/1.44×38 to +1.47/1.36×145	−2.73/1.46×37 to +1.30/1.39×146
Other retinal pathology	−2.67/1.45×39 to +1.42/1.35×145	−2.80/1.47×38 to +1.27/1.36×146
Previous vitrectomy surgery	−2.69/1.47×39 to +1.38/1.34×145	−2.84/1.47×38 to +1.24/1.34×146
Previous trabeculectomy surgery	−2.66/1.37×39 to +1.35/1.38×144	−2.82/1.39×38 to +1.18/1.40×145
Brunescent/white mature cataract	−2.65/1.45×38 to +1.41/1.35×145	−2.81/1.47×37 to +1.24/1.37×146
Posterior capsular rupture	−3.02/1.45×39 to +1.04/1.33×145	−3.18/1.47×38 to +0.90/1.34×146
Previous vitrectomy surgery and high myopia	−2.75/1.48×39 to +1.35/1.31×145	−2.91/1.50×38 to +1.20/1.32×146
Previous trabeculectomy surgery and glaucoma	−2.72/1.36×40 to +1.26/1.39×143	−2.88/1.38×39 to +1.12/1.39×144
Previous vitrectomy surgery and pseudoexfoliation/phacodonesis	−2.60/1.46×39 to +1.46/1.34×145	−2.76/1.46×38 to +1.30/1.36×146
Pseudoexfoliation/phacodonesis and glaucoma	−2.64/1.42×39 to +1.40/1.36×144	−2.79/1.42×38 to +1.24/1.37×145
Posterior capsular rupture and brunescent/white mature cataract	−3.02/1.46×39 to +1.06/1.31×145	−3.18/1.47×38 to +0.92/1.33×145
Posterior capsular rupture and pseudoexfoliation/phacodonesis	−2.95/1.44×39 to +1.11/1.33×145	−3.10/1.46×38 to +0.96/1.37×146
Posterior capsular rupture and high myopia	−3.10/1.48×39 to +1.01/1.30×145	−3.24/1.49×38 to +0.85/1.31×145
Posterior capsular rupture and uveitis/synaechiae	−3.06/1.48×39 to +1.04/1.33×145	−3.20/1.49×38 to +0.89/1.33×147

Results reported as compound numbers: sphere cylinder×axis.

*With a subjective refraction observed within 3 months of operation.

### Residual variance at the surgeon, patient and eye level

In our multilevel model the residual variance (ie, variance not explained by the covariates of the model) was partitioned into a between-surgeon component (the variance of the surgeon-level residuals), a between-patient component (the variance of the patient-level residuals) and a within-patient component (the variance of the eye-level residuals). The surgeon-level residuals represent the unobserved surgeon characteristics that affect the EERO (eg, whether the surgeon was left-handed or right-handed). The patient-level residuals represent the unobserved time-independent patient characteristics that might affect the EERO. The eye-level residuals represent the unobserved eye-level characteristics or time-dependent patient characteristics that affect the EERO, for example, changes in the thickness of the cataractous lens or the worsening of diabetes between operations on each eye.

Online supplemental appendix table 3 reports the random effects results of the cross-classified multilevel model (reported on the power matrix scale). We can use these random effects to understand how much of the residual variance in the outcome is attributed to (unobserved) differences between surgeons and between patients. In our final model (right-hand side of online supplemental appendix table 3), differences between surgeons and between patients explain only a small proportion of the residual variance. Differences between surgeons accounted for 4% of the residual variance in $f_{11}$ , 23% due to differences between patients, leaving 73% of the residual variance at the eye level. Similarly, for outcome $f_{22}$ . Note, for outcome $f_{12}$ , the model assumed no differences between patients (see online supplemental appendix for details), so 4% of the residual variance in $f_{12}$ was due to differences between surgeons, leaving 96% of the residual variance at the eye level.

**Figure 1:**
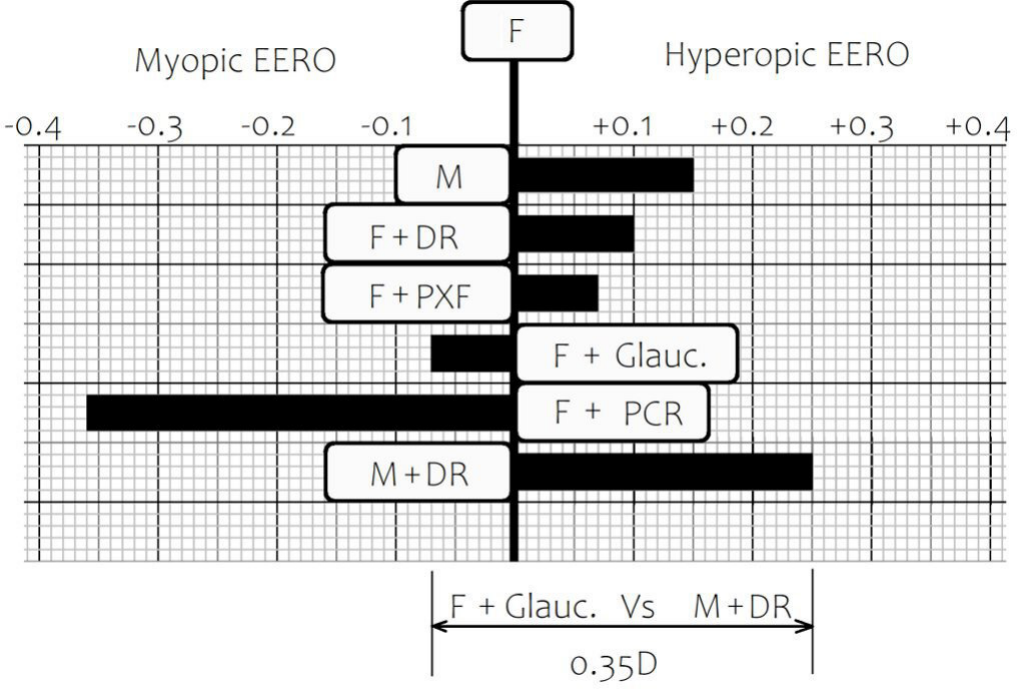
Error of expected refractive outcome (EERO) and co-morbidity. DR, diabetic retinopathy; F, women aged 77 years with no copathology unless otherwise specified; Glauc, glaucoma, M, men aged 77 years with no copathology unless otherwise specified; PCR, posterior capsule rupture; PXF, pseudoexfoliation/phacodonesis.

Adding covariates measured at the lowest level (in our case the eye level) to a model will always reduce the total amount of residual variance and the remaining variance at the lowest level.^28^ For example, adding covariates time since operation and refraction type reduced the total residual variance in $f_{11}$ , $f_{22}$ and $f_{12}$ from 1.23, 1.28 and 0.13, respectively, to 1.01, 1.09 and 0.12, respectively (compare the left-hand and right-hand results of online supplemental appendix table 3). Similarly, the eye level variances were smaller for the model including these covariates (eg, at the eye level, residual variance in $f_{11}$ decreased from 0.92 to 0.72). The variances at the patient and surgeon levels were virtually unaffected by the addition of covariates time since operation and refraction type, implying that the (within-patient and within-surgeon) distributions of these variables were similar across patients and surgeons, respectively.^28^ Although including eye-level factors refraction type and time of refraction since surgery reduced the residual variance at the eye level by 19%–23% for outcomes $f_{11}$ and $f_{22}$ , respectively, it had minimal impact on narrowing of the prediction intervals (results available on request). The online supplemental appendix also contains a discussion on how the missing data was managed.^29^ Figure 1 is a graphical representation of the impact of gender and/or a selection of comorbidities with respect to the refractive outcome.

## Discussion

The effect of the presence of ocular comorbidity has not previously been formally evaluated with respect to its impact on refractive outcomes, partly because of the difficulties in collecting a large enough sample to adequately power the statistical comparison. The adoption of electronic patient records by NHS trusts and the data collection for the National Ophthalmology Database has enabled the collection of 1 070 601 cataract operations, of which 490 989 cases could be analysed. This scale of data collection is unprecedented and has allowed identification of conditions which have contributed to refractive outcome following cataract surgery with a high level of precision. In this study, we used a cross-classified multilevel model to take into account the multilevel structure of the data (ie, operations nested within patients nested within surgeons and allowing a patient to receive two operations from different surgeons). This approach has enabled the assessment of the effect of surgeon, patient age, gender and comorbidity with very high precision. Using statistical methods that ignore the correct multilevel structure (eg, standard regression) will give SEs that are too small (ie, under-estimate the uncertainty), often leading to incorrect conclusions.^7^

The results of this study suggest that factors such as patient gender and comorbidity have a small but statistically and clinically significant impact on the EERO. For example, a male patient (mean EERO +0.12/0.05×91) with diabetic retinopathy (mean EERO +0.08/0.03×104) has a difference of +0.30/0.03×96 in refractive outcome than a female patient with glaucoma (mean EERO −0.10/0.05×95), which is almost as much as the mean EERO seen in posterior capsule rupture. To our knowledge, no IOL power formula considers the effect of comorbidities and very few of the currently used formulae take into account gender on the predicted refractive outcome, although the quantitative effect of gender used by these formulae has not been published. The effect of age was significant but its clinical significance is questionable as the difference was extremely small at −0.01/0.06×97 per decade. As described in the Results section, we considered the random effects (unexplained variance) at three levels: surgeon, patient and eye. The random effects at the surgeon level were minimal which indicates that there is little variation in EERO between surgeons and that most surgeons are undertaking similar surgery, that is, approximately 4% of the unexplained variance was due to unobserved differences between surgeons.

Hoffer and Savini reported improved refractive outcomes when taking into account gender and race but did not quantify the effect of gender.^30^ We found that men tended to have a more hyperopic EERO than women by +0.12/0.05×91. Women tend to have smaller eyes (axial length, corneal diameter and anterior chamber depth) than men and possibly these differences influenced the prediction accuracy of the IOL formulae used, although latest generation IOL power formulae which take into account more variables such as preop anterior chamber depth (ACD) and lens thickness still use gender as a variable that affects outcome,^31^ with men having a more myopic prediction than women.^32^ A number of copathologies were associated with small but statistically significant increases in the EERO such as diabetic retinopathy, pseudoexfoliation or phacodonesis, previous vitrectomy, high myopia, glaucoma or patients who had posterior capsular rupture. Diabetic retinopathy, pseudoexfoliation or phacodonesis were associated with hypermetropic astigmatic shift in EERO compared with eyes with no copathology or complication, while posterior capsule rupture, glaucoma, high myopia, previous vitrectomy were associated with myopic and astigmatic shift in EERO. Specifically, posterior capsule rupture was associated with an increased myopic and astigmatic error of −0.36/0.03×73, consistent with a more anteriorly placed IOL.^33^ Some of these increases in EERO could be expected. For example, patients with pseudoexfoliation have been reported to have deeper postoperative anterior chamber depths;^34^ conversely, patients with myopia have been reported to have negative prediction errors.^4^ The possible reasons behind the hyperopic shift in patients with diabetic retinopathy are less clear. Although some differences in biometric parameters such as shallower anterior chamber depths and thicker lenses have been reported in patients with type 1 diabetes, these changes have not been observed in patients with type 2 diabetes, which form the majority of patients.^35^ It is interesting to speculate that the difference may be due to a biometric bias as patients with diabetes with higher axial length/corneal radius ratios appear to have a lower risk of developing diabetic retinopathy^36^ so the relative hyperopic shift in patients with diabetic retinopathy could be argued to be simply due to an under-representation of eyes with longer axial lengths, thus resulting in a hyperopic prediction error.^4^ In our opinion, the absence of a small proportion of more myopic eyes from the diabetic retinopathy subgroup would be unlikely to result in such a high difference in the overall mean prediction error (+0.10D in nearest equivalent sphere).

Since their inception, IOL power formulae have been continuously improved by using better mathematical and physical models as well as the incorporation of additional and/or more precisely measured variables provided by modern biometry techniques. Initially, the improvements were very large but as formulae became more sophisticated, improvements in prediction error become progressively lesser in magnitude. As the effect of any remaining unknown factors/variables that contribute to residual prediction error becomes smaller, the number of eyes required to statistically power comparisons and quantify these factors increases. It appears that further progress and refinements in IOL power formulae will probably come about though many small refinements in both formula design and discovery of new variables. We believe that the incorporation of comorbidity and gender is one such step and it should be considered when developing and validating future IOL power formulae.

## Data Availability

Data may be obtained from a third party and are not publicly available. Data was provided by the National Ophthalmology Database at the Royal College of Ophthalmologists.
