# Supplementary material for: Surgeon effects on cataract refractive outcomes are minimal compared with patient comorbidity and gender: an analysis of 490 987 cases
Source: Br J Ophthalmol. 2021 Nov 11;107(4):488–94. doi: 10.1136/bjophthalmol-2021-320231 (PMC10086271; doi:10.1136/bjophthalmol-2021-320231)
Supplement: Supplementary data [file bjophthalmol-2021-320231supp001.pdf]

Appendix

We have demonstrated how to fit a multivariate two-way cross classified model to refractive data. A multivariate approach was necessary because we were interested in the simultaneous relationships between a covariate and the three components of the spherocylindrical refractive outcome. The multilevel model appropriately accounted for dependencies within the data and enabled investigation of how each level (e.g. surgeon, patient, operation) in the data contributed to the variance of the outcome. A key strength of multilevel modelling is its ability to accommodate a wide variety of multilevel structures and extensions to multivariate settings [26]. A downside of complex models, such as the multivariate cross classified model, is that they can be difficult to estimate using maximum likelihood methods [26]. However, with increasing computational power it is becoming easier to estimate such models using MCMC methods, which combined with diffuse priors can approximate maximum likelihood estimation. (23) Many different types of multilevel models are implemented in several popular software packages (e.g., Stata [27], SAS [28] and R[29]), although some of the more complex models can require specialised software such as MLwiN (25).

With regards to the missing data, we conducted a complete case analysis. This is a valid approach provided the chance of being missing does not depend on the outcome given the covariates of the model (e.g. complete case analysis would be invalid if those with a poor outcome were more likely to have missing data than those with a good outcome). Although this is not something we can directly test because we do not have access to the missing outcome values, we have carefully considered the reasons for missing data in our choice of analysis.

Most of the missing refractive data was due to patients seen in services where EMR data entry occurs in the operating theatre but not in postoperative outpatient clinics and so the main reason for missing data was independent of the underlying outcome values. We acknowledge that for a small percentage of patients, the chance of being included in our complete case analysis may depend on their missing outcome data. It is unlikely, however, that our results would be significantly affected by these few patients. Note, we did not conduct multiple imputation because we did not have access to auxiliary data (i.e. additional data outside of our analysis that explain the reasons for the missing data or provide information about the missing values). In the absence of auxiliary data, multiply imputing the outcome will not reduce bias nor gain information about the missing data, and could introduce bias due to miss-specification of the imputation model, and lead to larger standard errors due to multiple imputation’s inherent random process (29)

Appendix table 2 shows the fixed effects results of the (final) cross-classified model on the dioptric power matrix scale. The corresponding random effects results are reported in appendix table 3, with the left-half of the table reporting these results for the model excluding covariates time-period and type of refraction, and the right-half of the table reporting these results for the final model which included both of these covariates. Note, at the patient level, the variances and covariances involving outcome f_{12} were constrained to zero. In the unconstrained model these estimates were close to zero, and the constrained model was a better fit to the data than the unconstrained model (as measured by the deviance information criterion). The random effects results are a measure of the variance not explained by the model’s covariates. Including covariates for time period and refraction type reduced the unexplained variance at the operation level but had virtually no effect on the variance at the surgeon and patient levels.

Appendix table 1: Preference criterion used to select the single postoperative refraction measurement

Preference	Criterion categories
13 (Highest)	Subjective refraction measurement observed within one year of the operation
12	Autorefractive measurement observed within one year of the operation
11	Focimetry measurement observed within one year of the operation
10	Subjective refraction measurement observed more than one year since the operation
9	Autorefractive measurement observed more than one year since the operation
8	Focimetry measurement observed more than one year since the operation
7	Cycloplegic refraction measurement observed within one year of the operation
6	Retinoscopy refraction measurement observed within one year of the operation
5	Focimetry (2nd pair of glasses) measurement observed within one year of the operation
4	Cycloplegic refraction measurement observed more than one year since the operation
3	Retinoscopy refraction measurement observed more than one year since the operation
2	Focimetry (2nd pair of glasses) measurement observed more than one year since the operation
1 (Lowest)	Measurement of unknown type

Appendix table 2: Estimates of the fixed effects from the cross-classified multilevel model, reported on the dioptric power matrix scale.

	f_{11}		f_{22}		f_{12}	
	**Posterior mean (SD[$])**	**95% Credible interval**	**Posterior mean (SD)**	**95% Credible interval**	**Posterior mean (SD)**	**95% Credible interval**
Female aged 77 years[a]	-0.30 (0.0060)	-0.31, -0.29	0.15 (0.0061)	0.14, 0.16	-0.033 (0.0020)	-0.037, -0.029
Male	0.17 (0.0030)	0.17, 0.18	0.12 (0.0032)	0.11, 0.13	0.00052 (0.00098)	-0.0014, 0.0024
Age (per 10 years)	0.046 (0.0016)	0.043, 0.049	-0.0085 (0.0016)	-0.012, -0.0053	0.0071 (0.00051)	0.0060, 0.0080
Diabetic retinopathy	0.11 (0.0064)	0.098, 0.12	0.084 (0.0067)	0.071, 0.097	0.0071 (0.0021)	0.0030, 0.011
Pseudoexfoliation/ Phacodonesis	0.067 (0.015)	0.038, 0.096	0.077 (0.015)	0.047, 0.11	0.0049 (0.0050)	-0.0049, 0.015
Previous vitrectomy surgery	-0.014 (0.012)	-0.037, 0.0085	-0.034 (0.012)	-0.058, -0.010	-0.011 (0.0040)	-0.018, -0.0029
Previous trabeculectomy surgery	-0.016 (0.023)	-0.061, 0.030	-0.066 (0.024)	-0.11, -0.018	0.025 (0.0078)	0.0098, 0.040
High myopia	-0.045 (0.0072)	-0.060, -0.031	-0.058 (0.0075)	-0.072, -0.043	-0.013 (0.0024)	-0.018, -0.0085
Glaucoma	-0.050 (0.0054)	-0.061, -0.040	-0.099 (0.0056)	-0.11, -0.088	0.0043 (0.0018)	0.00079, 0.00077
Posterior capsular rupture	-0.035 (0.014)	-0.38, -0.32	-0.38 (0.015)	-0.41, -0.35	-0.011 (0.0050)	-0.021, 0.0010
Uveitis/synaechiae	-0.026 (0.017)	-0.060, 0.0077	0.0023 (0.018)	-0.033, 0.037	-0.0088 (0.0058)	-0.020, 0.0026
Other retinal pathology	0.024 (0.015)	-0.0055, 0.053	0.00059 (0.015)	-0.030, 0.031	-0.0057 (0.0051)	-0.016, 0.0044

Continued on next page

	f_{11}		f_{22}		f_{12}	
	**Posterior mean (SD[$])**	**95% Credible interval**	**Posterior mean (SD)**	**95% Credible interval**	**Posterior mean (SD)**	**95% Credible interval**
Brunescent/white mature cataract	0.014 (0.0074)	-0.0010, 0.029	0.0045 (0.0077)	-0.011, 0.020	-0.0046 (0.0025)	-0.0095, 0.00032
Time period						
within 3 months[b]	-	-	-	-	-	-
between 3 and 6 months	0.065 (0.0054)	0.055, 0.076	0.090 (0.0057)	0.079, 0.10	0.010 (0.0019)	0.0066, 0.014
between 6 and 12 months	0.086 (0.0072)	0.072, 0.10	0.11 (0.0075)	0.10, 0.13	0.0095 (0.0025)	0.0046, 0.014
more than 12 months	0.12 (0.0080)	0.11, 0.14	0.18 (0.0083)	0.16, 0.19	0.0075 (0.0027)	0.0021, 0.013
Refraction type						
subjective[b]	-	-	-	-	-	-
autorefractive	-0.056 (0.0042)	-0.064, -0.048	-0.013 (0.0044)	-0.022, -0.0048	-0.017 (0.0014)	-0.019, -0.014
focimetry	0.019 (0.0065)	0.0057, 0.031	0.020 (0.0067)	0.0071, 0.033	0.000051 (0.0022)	-0.0042, 0.0043
focimetry (2nd pair of glasses)	0.0080 (0.025)	-0.041, 0.057	0.034 (0.026)	-0.016, 0.086	-0.016 (0.0085)	-0.033, 0.00037
other	-0.15 (0.021)	-0.19, -0.11	-0.12 (0.022)	-0.16, -0.077	0.011 (0.0069)	-0.0026, 0.025

SD: Standard Deviation. a: Reference patient (woman aged 77 years with a subjective refraction observed within 3 months of operation). b Reference category of that variable.

Appendix table 3: Estimates of the random effects from the cross-classified multilevel model, reported on the dioptric power matrix scale. Results reported for a model that excludes and includes covariates for the type of refraction and the time period of the post-operative refraction

	**Model excluding covariates time period and type of post-operative refraction**			**Final model including covariates time period and type of post-operative refraction**		
	**Posterior Mean**	**Standard Deviation**	**95% Credible Interval**	**Posterior Mean**	**Standard Deviation**	**95% Credible Interval**
Surgeon level						
$Var(f_{11})$	0.045	0.0020	0.041, 0.049	0.046	0.0020	0.042, 0.050
$Cov(f_{11}, f_{22})$	0.032	0.0018	0.028, 0.035	0.031	0.0018	0.028, 0.035
$Var(f_{22})$	0.047	0.0021	0.043, 0.051	0.046	0.0021	0.042, 0.050
$Cov(f_{11}, f_{12})$	0.0017	0.00046	0.00085, 0.0026	0.0017	0.00045	0.00085, 0.0026
$Cov(f_{22}, f_{12})$	-0.0027	0.00047	-0.0035, -0.0017	-0.0027	0.00046	-0.0036, -0.0018
$Var(f_{12})$	0.0050	0.00021	0.0046, 0.0054	0.0051	0.00021	0.0047, 0.0055
Patient level						
$Var(f_{11})$	0.26	0.0034	0.25, 0.27	0.24	0.0028	0.23, 0.25
$Cov(f_{11}, f_{22})$	0.19	0.0029	0.18, 0.19	0.17	0.0024	0.17, 0.18
$Var(f_{22})$	0.27	0.0034	0.26, 0.28	0.26	0.0029	0.25, 0.26
Eye level						
$Var(f_{11})$	0.92	0.0034	0.91, 0.92	0.72	0.0027	0.72, 0.73
$Cov(f_{11}, f_{22})$	0.64	0.0030	0.63, 0.64	0.47	0.0024	0.47, 0.48
$Var(f_{22})$	0.96	0.0035	0.95, 0.97	0.78	0.0029	0.78, 0.79
$Cov(f_{11}, f_{12})$	0.0069	0.00053	0.0058, 0.0079	0.0065	0.00046	0.0056, 0.0074
$Cov(f_{22}, f_{12})$	-0.0013	0.00054	-0.0023, -0.00021	-0.0020	0.00048	-0.0030, -0.0011
$Var(f_{12})$	0.12	0.00024	0.12, 0.12	0.11	0.00023	0.11, 0.11

Hughes R, *et al. Br J Ophthalmol* 2023; 107:488–494. doi: 10.1136/bjophthalmol-2021-320231
